# Origami Inspired Laser Scanner

**DOI:** 10.3390/mi13101796

**Published:** 2022-10-21

**Authors:** Yu-Shin Wu, Shao-Kang Hung

**Affiliations:** Department of Mechanical Engineering, National Yang Ming Chiao Tung University, No. 1001, University Road, Hsinchu 30010, Taiwan

**Keywords:** origami, laser scanner, galvanometer, mechatronics

## Abstract

Diverse origami techniques and various selections of paper open new possibilities to create micromachines. By folding paper, this article proposes an original approach to build laser scanners, which manipulate optical beams precisely and realize valuable applications, including laser marking, cutting, engraving, and displaying. A prototype has been designed, implemented, actuated, and controlled. The experimental results demonstrate that the angular stroke, repeatability, full scale settling time, and resonant frequency are 20°, 0.849 m°, 330 ms, 68 Hz, respectively. Its durability, more than 35 million cycles, shows the potential to carry out serious tasks.

## 1. Introduction

Laser scanners, also known as galvanometers, utilize motor-driven mirrors to reflect a laser spot to a desired location accurately and rapidly. By repeating the process along a scheduled trajectory, the focused point burns into the target surface and performs laser machining such as marking, cutting, and engraving [1]. Laser galvanometers are also used in modern technologies including lidar [2,3,4], stereolithography [5], selective laser sintering/melting [6], laser scanning display [7,8,9,10,11], optical coherence tomography [12], and scanning laser ophthalmoscopy [13,14]. In brief, these useful devices are widely deployed in various fields.

Regarding the inner structure, comprehensively discussed in literatures [15,16,17,18], a laser galvanometer consists of a mirror, an angular actuator, and control electronics. The mirror needs high reflectance and low inertia. The angular actuator is expected to possess high resolution, wide stroke, and fast response. The controllers are divided into open-loop and closed-loop types with typical examples [1,19], which are mature products available in the market. Compared in Table 1, we have found various designs to meet the different requirements. For example, manufacturing tools need fast response; medical instruments rely on accuracy; and consumer devices demand cost-effectiveness. Therefore, every specific design has its value.

Origami, an ancient art inherited from the orient, has been re-purposed as modern engineering applied in aerospace [20], stents [21,22], solar panels [23], haptic devices [24], and robots [25,26,27,28]. The properties of one-fold hinge have been investigated [29], and well-designed multiple folds become joints with several degree-of-freedoms [30]. Origamizer [31] is an algorithm that allows every polyhedral complex to be folded from a sufficiently large paper. Since so much knowledge about origami has been accumulated, origami-inspired machines can now be created to do practical work. This article proposes a revolutionary origami-based method for making laser galvanometers.

Regarding the advantages of the proposed method, rotary joints are classified into 3 types: Ball bearings, torsional beams [15,16,17,18,32], and origami hinges of this work. Ball bearings can rotate endlessly and have the widest scanning range, but the friction degrades their accuracy or makes them hard to be controlled. Torsional beams, thanks to their frictionless elastic deformation, can rotate accurately within a narrow range. Origami hinges act as an in-between, which offer both frictionless motion and enough scanning range for the applications of laser galvanometers. Furthermore, origami hinges are extraordinarily cost-effective and can be easily manufactured by a desktop laser cutter.

The structure of this paper is organized as follows. Section 2 introduces the system design including kinematic simulations, actuators, and sensors. Section 3 describes instrumentations and experiments. Section 4 presents the conclusion of this article and improvements that can be carried out in the future.

## 2. Design

From Table 1, the main specifications of a miniature laser engraver [1] are ±10° mirror scanning range, 0.04° repeatability, and 10 s settling time of full scale. We hope to make a competitive one with a much lower cost. Therefore, the goals of this work are set to the same ±10° range, better repeatability, 10-time faster settling time, and 10-time more operational cycles.

### 2.1. Paper Mechanism

Intuitively, a straightforward fold transforms a piece of paper into two linkages with a connecting joint. One linkage is fixed as the ground, and the other linkage carries the mirror rotating around the folded line. We have tested this concept and found a fatal drawback: long-lasting oscillation such as a thin cantilever. Obviously, this is not a good idea.

**Figure 1:**
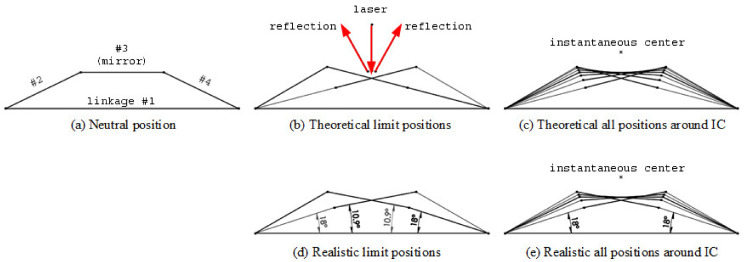
(**a**) Neutral position, (**b**) limit positions, and (**c**) kinematic diagram of the theoretical four-bar mechanism. (**d**) Limit positions and (**d**) kinematic diagram of the realistic four-bar mechanism with 18° hard stopper to avoid mechanical singularity.

With the above experience, we choose four-bar mechanism with one degree of freedom as well. Illustrated in Figure 1a, linkage #1 is fixed as the ground, linkage #2 and #4 are the rockers, and linkage #3 is the mirror carrier with connecting joints at both ends. Since the mirror is not supported at only one end, the aforementioned drawback can be significantly suppressed. The dynamic characteristics will be quantified in experiments.

Figure 1b shows the theoretical limit positions of this four-bar mechanism. The mirror at the rightmost position reflects the incident laser to the leftmost direction. In the same manner, when the mirror turns to the opposite side, the laser is reflected to the leftmost direction. According to the law of reflection, the scanning range of the reflected laser is the double of the swinging range of the mirror.

The full motion of this four-bar mechanism is shown in Figure 1c. Linkage #3 approximately rotates around the instantaneous center (IC) [33], located at the intersection point of the extensive lines of linkage #2 and #4 at their neural position. In summary, a virtual hinge at IC has been created, and the mirror rotates around it smoothly. Controlling this four-bar mechanism implies manipulating the reflected laser beam. The next step is to determine the length values of every linkage.

**Figure 2:**
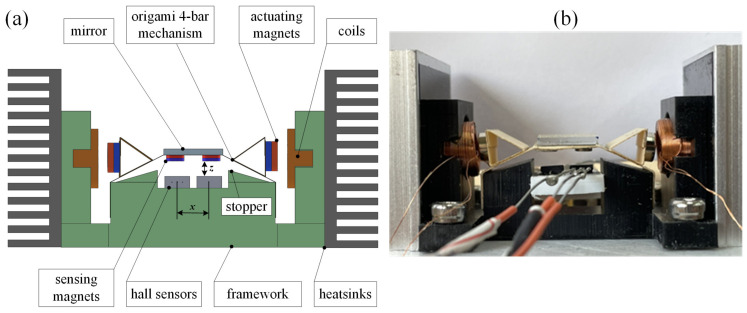
(**a**) Schematic diagram and (**b**) photograph of the proposed origami laser galvanometers.

Since the projected laser spot is an 8 mm × 5 mm ellipse [1,34], the mirror size has to be a little bit larger to cover the whole spot and is set to a 10 mm × 10 mm square. Therefore, the length of linkage #3 is 10 mm. Secondly, to perform symmetrical scanning motion, linkages #2 and #4 must be equal in length. According to our experience of implementation, 10 mm is also a suitable choice, too. Next, the mechanical singularity [35] condition, i.e., linkages #2 and #3 are collinear, has to be avoided; therefore, the realistic limit positions are set as Figure 1d. That means an external 18° hard stopper, labeled in Figure 2a, should be adopted to prevent the singular condition. Accordingly, the length of linkage #1 is 28 mm, and the mirror scanning range is ±10.9°, which is slightly wider than we demand. This tolerance is meaningful to absorb misalignment in following experiments. Finally, the kinematic diagram is shown in Figure 1e.

### 2.2. Actuators

Electromagnetic actuators are chosen in this work similar to most galvanometers. Intuitively, magnets can be directly attached onto the rocker linkages, and then the coils push/pull the magnets and drive the apparatus. To dissipate heat, however, coils need big heat sinks, which may interfere with the optical path. Therefore, we add a triangle structure to extend the rocker linkages as shown in Figure 2. Strong NdFeB magnets with 5 mm diameter are glued on the vertical surfaces. Coils are installed aside with heatsinks.

### 2.3. Sensors

**Figure 3:**
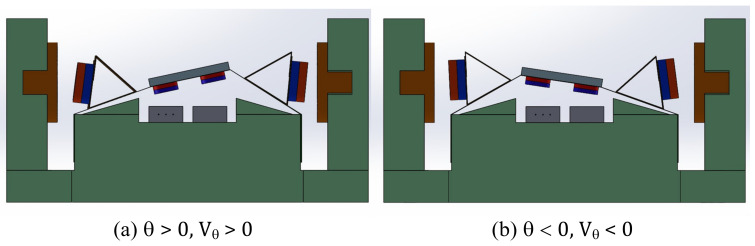
(**a**) Positive and (**b**) negative tilting angles with a corresponding sensor signals.

Under the mirror, two smaller magnets with 3 mm diameter are attached with a central distance of 6 mm. When the mirror is rotating, the changing magnetic flux affects underneath hall sensors (SS49E, Honeywell), which are installed correspondingly. We define the differential voltage between two hall sensors as Equation (1) to present the tilting angle of the mirror, where $V_{RH}$ and $V_{LH}$ are the signals of the right and the left hall sensors, respectively. Figure 3 defines the positive direction of the mirror’s rotation.

(1) $$V_{\theta }=V_{RH}-V_{LH}$$

**Figure 4:**
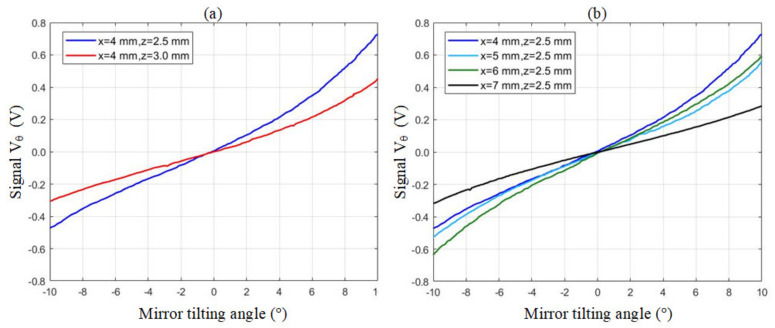
(**a**) The same magnet-sensor gap with different sensor-sensor distance. (**b**) The same sensor-sensor gap with different magnet-sensor gap.

Furthermore, the installation position of hall sensors should be optimized. Recalling Figure 2, the neutral position, *z* is the gap between the sensing magnet and the hall sensor below. To avoid collision, *z* must be greater than 2 mm. The influence of variable z has been tested and plotted in Figure 4a. The results show that, for a fixed x value, a smaller z value contributes a wider dynamic range of the output signal because of the strong magnetic field near the surface of the magnet. Therefore, we set z equal to 2.5 mm, the practically shortest distance before collision happens.

In addition, the central distance between two hall sensors, x, should be taken into consideration, too. To avoid collision, *x* must be greater than or equal to 4 mm. The influence of variable x has been plotted in Figure 4b. The conditions of x = 4 mm and 7 mm are dropped off due to poor symmetricity and low sensitivity, respectively. Finally, we choose the condition of x = 5 mm because of its balanced symmetricity and wide dynamic range. All design parameters are summarized in Table 2. With detailed technical data [36], the thickness and the areal density of our selected paper (Pop’Set, Arjowiggins) in this work are 0.21 mm and 170 g/m^2^, respectively. Naturally, the material of paper affects the properties of the proposed system. Thicker/denser paper leads to stiffer hinges, higher resonant frequency, and more current consumption for maintaining the same titling angle.

**Figure 5:**
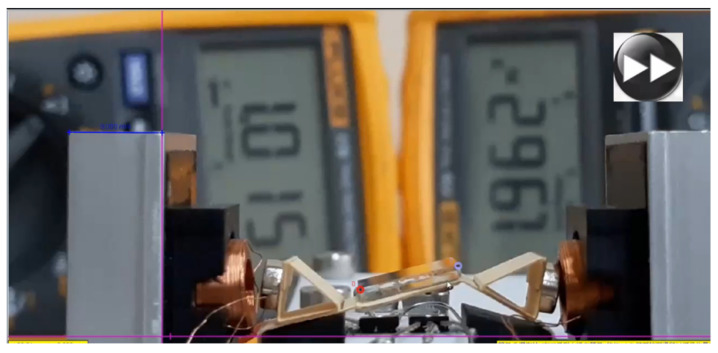
A snapshot captured by Tracker program, which analyses the motion of the tilting mirror.

Aforementioned mirror titling angle is measured by an open-source program, Tracker [37], which analyses the videos by image processing algorithms. A clip of motion videos is demonstrated [38]. As a snapshot captured in Figure 5, two black points are marked at two corners of the mirror as the “feature points”. Tracker has the ability to identify these feature points in the video stream and calculate the positions and the associated tilting angle. The background of the motion video also records the voltages of hall sensors. Now, the relationship between the sensors’ signals and the mirror tilting angle can be well mapped, as plotted as the cyan line in Figure 4b. In the following experiments, we rely on $V_{\theta }$ to measure the mirror tilting angle because hall sensors act much faster than our visual camera, whose acquisition rate is limited at 30 frame/s.

## 3. Experiment

### 3.1. Instrument Setup

**Figure 6:**
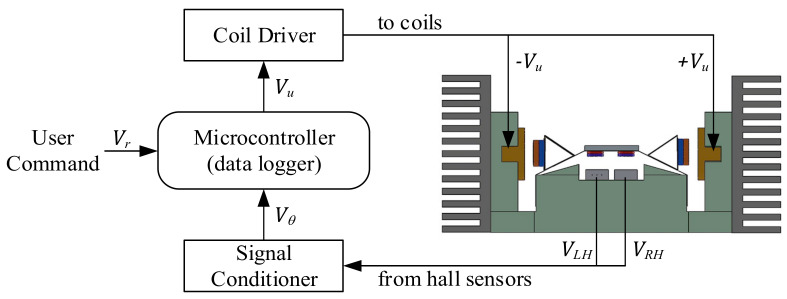
The block diagram the proposed system.

A general-purpose microcontroller (ESP32, Expressif) plays the role of the system integrator in Figure 6. The signal conditioner is implemented by operational amplifiers (TL084, Taxes Instruments Texas Instruments). After proper amplifying and offsetting, the hall sensors’ signals are acquired by the analog input channels of the microcontroller. The control program delivers calculated output signals to the 2-channel coil driver (L298N, STMicroelectronics) and generates push-pull electromagnetic force to manipulate the four-bar mechanism. The mirror’s angle will be read back again to complete a closed-loop. A series of experiments have been conducted to demonstrate the performance of this system in the next section.

### 3.2. Durability Test

**Figure 7:**
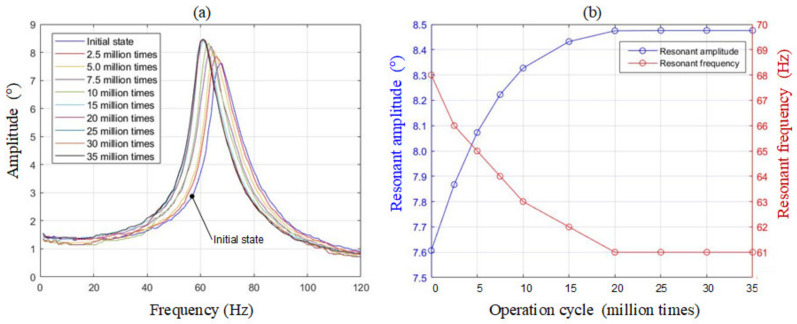
(**a**) Spectrums of the proposed system. (**b**) Origami hinges are softened along with increasing operation cycles.

Before going further, we must ask questions such as “Is a paper-made machine durable?” and “How many cycles it can work for?” Therefore, an accelerated aging exam has been conducted in the following procedures. Initially, by scanning the spectrum at a constant coil voltage, we find the resonant frequency and the magnitude are 68 Hz and 7.6°, respectively. Next, the proposed system is driven at resonance for 2.5 million cycles, i.e., 613 min, and then rescanned again to record new values of resonant frequency and the magnitude. The whole process is automatically repeated for 35 million cycles in total. The results are plotted in Figure 7.

**Figure 8:**
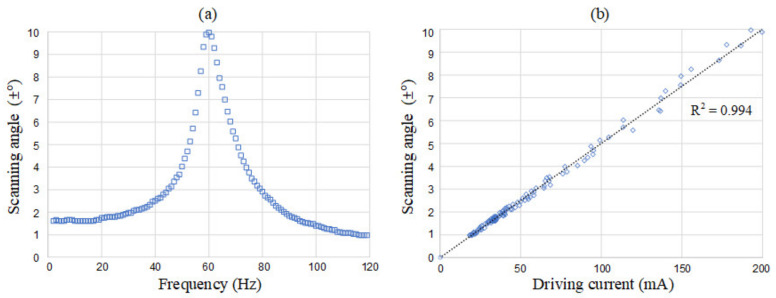
Characterization of the origami galvanometer. (**a**) Scanning angle versus driving frequency. (**b**) Scanning angle versus driving current.

In the first 20 million cycles, the resonant frequency decreases from 68 Hz to 61 Hz, and the magnitude increases from 7.6° to 8.47° gradually. In the final 15 million cycles, both resonant frequency and magnitude remain unchanged. The results show that the motion may “soften” the origami hinges in the early stage, and then the device becomes stable with constant properties. The minimal lifetime is 35 million cycles, which meets our goals. This aged device is used in the following experiments and tested in Figure 8a. Now we increase the driving voltage and keep the resonant amplitude less than ±10° limit, or the device may hit the hard stopper. The relationship between the scanning angle and the driving current is plotted in Figure 8b.

### 3.3. Classic PD Control

**Figure 9:**
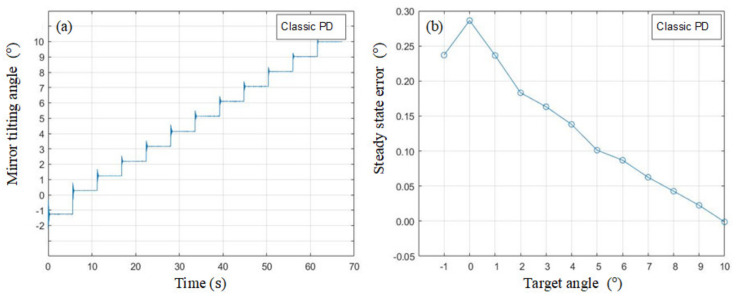
(**a**) Step-train response and (**b**) steady state error of the classic PD controller.

Since we know the mechanical properties may drift, the system cannot be well controlled only by an open loop. We adopt the classic proportional-differential (PD) feedback control scheme. The first controller is expressed as Equations (2) and (3);

(2) $$e=V_{r}-V_{\theta }$$

(3) $$V_{u}=K_{O}V_{r}+K_{P}e+K_{D}\dot{e}$$

where *e* is the error between the user command and the sensor feedback. *Vu* is the control output combined by the open-loop, proportional, and differential parts. The open-loop gain $K_{O}$ is 0.5 adjusted by a full-range swing pretest. P-gain $K_{P}$ and D-gain $K_{D}$ are 275 and 8.5, respectively. The step-train response and the corresponding steady state error are plotted in Figure 9a,b, where there is nonnegligible steady state error. Therefore, we need a more advanced control scheme.

### 3.4. Variable Gain PID Control

**Figure 10:**
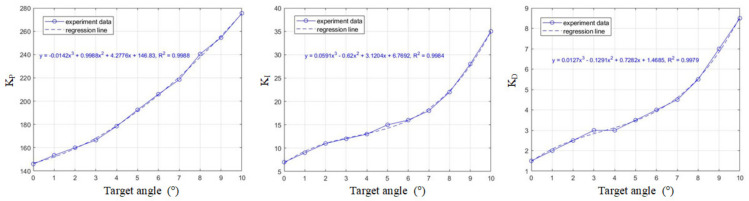
Control gains are adaptively changed with the target angle.

**Figure 11:**
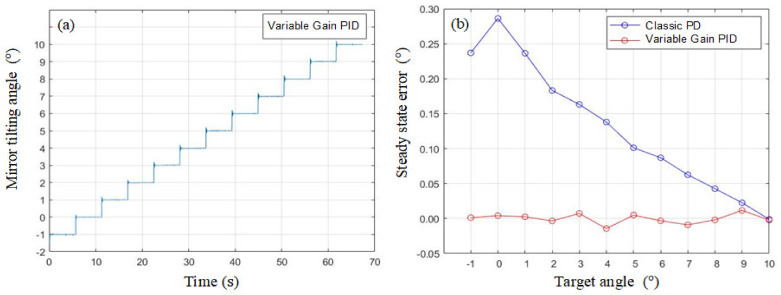
(**a**) Step-train response and (**b**) steady state error of the variable gain PID controller.

Theoretically, integral control can suppress steady state error and provide accuracy. Any residual error will be accumulated to compensate the system until the error approaches zero. Now the controller is reformed as Equation (4);

(4) $$V_{u}=K_{O}V_{r}+K_{P}e+K_{I}\int edt+K_{D}\dot{e}$$

where the additional parameter $K_{I}$ is the integral gain. In Figure 10, $K_{P}$, $K_{I}$, and $K_{D}$ gains are adaptively changed with the target angle to compensate the nonlinearity of the system. The step-train response and the corresponding steady state error are plotted in Figure 11a,b, where the steady state error has been suppressed significantly. Since the behavior of the negative target angle is similar to the positive target angle due to the symmetrical structure, only the positive half plans are plotted.

**Figure 12:**
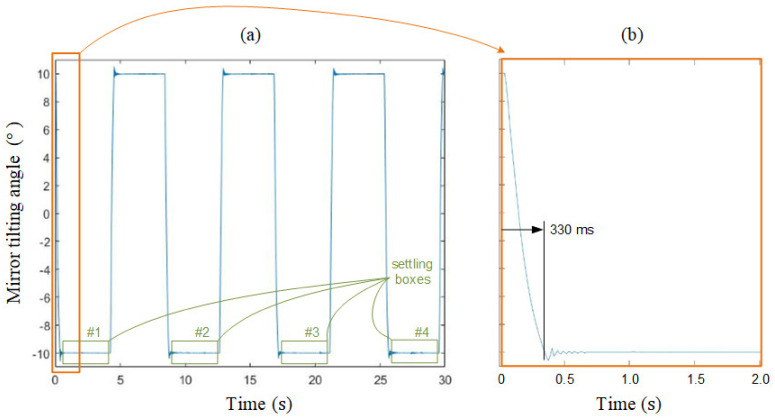
(**a**) Full scale regulation response of the proposed system. (**b**) The detailed view of settling time.

After obtaining a satisfactory controller, the next experiment is the full-scale regulation. The results are plotted in Figure 12a, which proves the scanning range of the proposed device is ±10°. The region of the beginning two seconds is zoomed in Figure 12b to specify the full-scale settling time, 330 ms. After settling, the mirror tilting angle is supposed to be −10° theoretically; however, there is experimental fluctuation. We record the data inside each “settling box” #1 to #10, and then calculate their standard deviation, 8.49 × 10^−4^°, to represent the positioning repeatability. In summary, above experimental results satisfy the goals we set in Table 1.

## 4. Discussion

In Figure 7a, from DC to 20 Hz, there is a flat region with a constant amplitude. Therefore, we set 20 Hz is the operational limit, which fully satisfies our speed goal in Table 1. This operational limit is separated from the first resonant frequency, 61 Hz, and higher resonant frequencies of other modes. This limitation implies stability theoretically. On the other hand, in Figure 12, the ±10° rapid swinging response also validate the stability and the accuracy of the proposed system experimentally. Noteworthily, if the paper mechanism hits the hard stopper, rebound will occur and induce unstable oscillations. In summary, over-driving should be prevented.

Compared with traditional laser scanning systems such as galvanometers [19], origami hinges provide frictionless pivots; thus, the actuating and controlling efforts are lower. Microelectromechanical systems (MEMS) galvanometers had been reviewed thoroughly by Holmström et al. [39]. Compared with common MEMS galvanometers in his study, our origami galvanometer has a much larger mirror size, which is capable to withstand high laser power for machining tasks. On the contrary, MEMS galvanometers suit for relatively lower laser power, higher frequency, and multi-pixel applications such as lidar and image projection display. In addition, our origami galvanometers demonstrate clear advantages including effective cost, easy fabrication, and short developing period.

**Figure 13:**
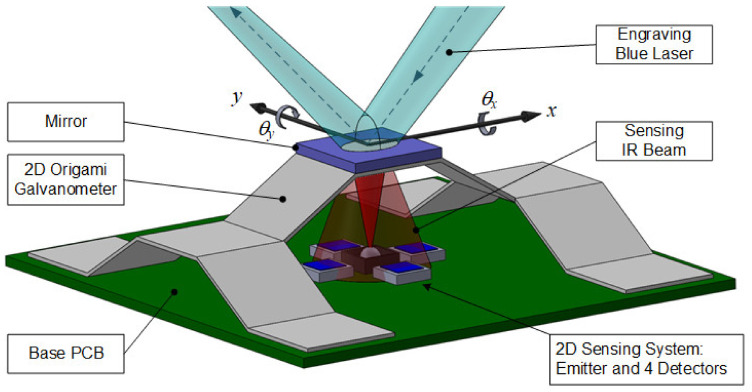
The concept of the 2D origami galvanometer with an optical sensing system.

A realistic laser engraver or image displayer demands two dimensions (2D). The idea of this work can be extended to a 2D version as illustrated in Figure 13. Coils work in the same manner but are not shown in order to view the internal structure clearly. Several trapezoidal 4-bar mechanisms are stacked orthogonally to tilt the mirror around x- and y- axes. A 2D optoelectronic angular sensor [34], our former work, can be seamlessly integrated.

## 5. Conclusions and Outlook

This article presents the first laser galvanometer built using origami techniques. A paper-folded four-bar mechanism has been designed for tilting the mirror around its instantaneous center. Electromagnetic actuators and hall sensors are integrated as a mechatronic system. Classic PD scheme and variable gain PID scheme have been implemented to control the proposed device successfully. The experimental results demonstrate that the angular stroke, repeatability, full scale settling time, and resonant frequency are 20°, 0.849 m°, 330 ms, 61–68 Hz, respectively. Its durability, more than 35 million cycles, meets the requirements for a miniature laser engraver.

In the future, we will attempt more parameters including various paper materials, thickness, and surface coatings. In general, the material determines durability; the thickness affects the stiffness of hinges; and the surface coating may prevent the influence of humidity. In addition, upgrading to a two-dimensional optoelectronic sensor will help to construct a laser engraving system without image distortion [40]. This article emphasizes the novelty of the paper-made mechanisms, but not controllers. We are planning to identify the dynamics of the origami systems and develop a specialized control scheme for them. Furthermore, more complicated designs with multiple degree-of-freedom will be a hopeful prospect.

## Figures and Tables

**Table 1:** Main specifications of typical 10 mm aperture laser galvanometers.

	[19]	[1]	Goals of This Work
Recommended aperture	10 mm	10 mm	10 mm
Actuator type	DC motor	Stepping motor	Voice coil motor
Bearing type	Ball bearing	Ball bearing	Origami hinges
Controller type	Closed-loop	Open-loop	Closed-loop
Scan range	±12°	±10°	±10°
Repeatability (rms)	5.73 × 10^−5^ degree	0.04 degree	≤0.004 degree
Settling time	0.25 ms (for 1% of full scale)	10 s (for full scale)	≤1 s (for full scale)
Durability	Not available	1.577 million cycles *	≥15.77 million cycles
Cost	Very high	Medium	Low
Recommended aperture	10 mm	10 mm	10 mm
Actuator type	DC motor	Stepping motor	Voice coil motor

* calculated by 24 h/day full time operation during 1-year warranty.

**Table 2:** Design parameters of the proposed origami laser galvanometer.

Design Parameters	Values
Paper: thickness and areal density	0.21 mm, 170 g/m^2^
Mirror: weight and size	0.7 g, 10 × 10 × 1.2 mm^3^
4-bar mechanism: length values	28 mm, 10 mm, 10 mm, 10 mm
Driving magnets: size and surface flux density	φ 5 mm × 2 mm, 300 mT
Sensing magnets: size and surface flux density	φ 3 mm × 1 mm, 220 mT
Magnet-sensor gap	2.5 mm
Sensor-sensor distance	5 mm

## Data Availability

Not applicable.
